# Direct association of visit-to-visit HbA1c variation with annual decline in estimated glomerular filtration rate in patients with type 2 diabetes

**DOI:** 10.1186/s40200-015-0201-y

**Published:** 2015-09-14

**Authors:** Akiko Takenouchi, Ayaka Tsuboi, Mayu Terazawa-Watanabe, Miki Kurata, Keisuke Fukuo, Tsutomu Kazumi

**Affiliations:** Postgraduate School of Food Sciences and Nutrition, Nishinomiya, Japan; Department of Food Sciences and Nutrition, School of Human Environmental Sciences, Nishinomiya, Japan; Research Institute for Nutrition Sciences, Mukogawa Women’s University, 6-46, Ikebiraki-cho, Nishinomiya, Hyogo 663-8558 Japan; Diabetes Division, Sadamitsu Hospital, Kakogawa, Hyogo 675-0005 Japan

**Keywords:** HbA1c, Standard deviation, Kidney function, eGFR

## Abstract

**Background/Aims:**

This study examined associations of visit-to-visit variability of glycemic control with annual decline in estimated glomerular filtration rate (eGFR) in patients with type 2 diabetes attending an outpatient clinic.

**Methods:**

Intrapersonal mean and coefficient of variation (CV) of 8-12 measurements of HbA1c and those of 4-6 measurements of fasting and post-breakfast plasma glucose (FPG and PPG, respectively) during the first 12 months after enrollment were calculated in a cohort of 168 patients with type 2 diabetes. Annual changes in eGFR were computed using 52 (median) creatinine measurements obtained over a median follow-up of 6.0 years. Multivariate linear regressions assessed the independent correlates of changes in eGFR.

**Results:**

CV-HbA1c (standardized β、-0.257、*p =* 0.004) were significantly and log urine albumin/creatinine ratio (standardized β、-0.155、*p =* 0.085) and smoking (standardized β、-0.186、*p =* 0.062) tended to be associated with annual eGFR decline independently of mean HbA1c, age, sex, BMI, waist circumference, diabetes duration and therapy, means and CVs of FPG, PPG and systolic blood pressure, baseline eGFR, and uses of anti-hypertensive and lipid-lowering medications. Association between HbA1c variability and renal function decline was stronger in patients with albumin/creatinine ratio ≧ 30 mg/g than in those with normoalbuminuria (r = -0.400, *p =* 0.003 and r = -0.169, *p =* 0.07, respectively).

**Conclusions:**

Consistency of glycemic control is important to preserve kidney function in type 2 diabetic patients, in particular, in those with nephropathy.

## Background

Diabetes is an important cause of mortality and morbidity worldwide, through both direct clinical sequelae and increased mortality from cardiovascular and kidney diseases [1]. Long-term glycemic control, as expressed by hemoglobin (Hb) A1c levels, is the main risk factor for the development of microvascular complications including diabetic kidney disease [2, 3]. Among patients with diabetes mellitus, elevated blood pressure (BP) is associated with progression of microvascular complications such as nephropathy and retinopathy [4]. In addition to high BP and hyperglycemia, dyslipidemia has an important role in the progression of kidney disease in patients with diabetes [5].

There is emerging interest to examine the influence of glycemic and BP variance in diabetic vascular complications [6, 7]. Recently, variation of HbA1c, a reflection of long-term glycemic fluctuation, was found to increase the risk of renal and cardiovascular complications [8–17]. In all studies on renal complications (8–10, 12–17), researchers focused on the relation between HbA1c variability and development and/or progression of diabetic nephropathy. Direct association between HbA1c variability and changes in kidney function has hardly been investigated. We, therefore, asked the question whether HbA1c variability might directly associated with annual decline in estimated glomerular filtration rate (eGFR) in patients with type 2 diabetes attending a long-term follow-up in a single outpatient clinic.

## Methods

The setting for this observational study was an outpatient diabetes clinic in a private hospital in Japan. Study protocol was consistent with the Japanese Government’s Ethical Guidelines Regarding Epidemiological Studies in accordance with the Declaration of Helsinki. We examined a cohort of 168 patients with type 2 diabetes who had been regularly attending the clinic in 2004 and 2005. They were enrolled in the study at the first visit in 2005 and had at least 8 monthly visits with blood samplings during the first 12 months after enrollment. Of 168 patients, 153 patients (91 %) had 12 monthly visits with blood samplings. In the 153 patients, blood was withdrawn on 2 occasions; at 2 h after breakfast taken at home and after an overnight fasting. This was done every other month. In the remaining 15 patients, blood was obtained after an overnight fasting. The main clinical features of these subjects at baseline are reported in Table 1.

**Table 1:** Anthropometric, clinical and biochemical features of 168 patients with type 2 diabetes and correlation coefficients of annual changes in estimated glomerular filtration rate and coefficients of variation of HbA1c

	Mean ± SD or n, %			⊿eGFR		CV-HbA1c	
Male sex (n, %)	90	,	54	−0.013		−0.17	*
Smokers (n, %)	58	,	34	−0.159	*	0.111	
Age (years)	62.3	±	10	0.037		−0.145	
BMI (kg/m^2^)	24.2	±	3.6	−0.048		0.045	
Waist circumference (cm)	86.9	±	9.9	−0.108		0.017	
Duration of diabetes (years)	9.9	±	7.3	−0.047		−0.009	
Treatment of							
diabetes; diet/OHA/insulin (%)	31/51/18			−0.078		0.201	**
hypertension; CCB/RASi/diuretics (%)	34/41/5			−0.076		−0.044	
HbA1c (%)	7.0	±	0.8	−0.050		0.343	***
Fasting PG (mg/dL)	125	±	22	−0.012		0.299	***
Post-breakfast PG (mg/dL)	154	±	49	0.047		0.229	**
CV-HbA1c (%)	7.0	±	6.4	−0.187	*	1	
CV-Fasting PG (%)	14.1	±	9.3	−0.127		0.473	***
CV-Post-breakfast PG (%)	21.9	±	11.0	−0.152		0.190	*
Total cholesterol (mg/dL)	188	±	21	0.048		0.025	
LDL cholesterol (mg/dL)	111	±	22	0.0004		0.096	
HDL cholesterol (mg/dL)	56	±	15	0.128		−0.202	**
Fasting TG (mg/dL)	115	±	51	−0.161	*	0.187	*
Post-breakfast TG (mg/dL)	145	±	64	−0.164	*	0.235	**
Serum creatinine (mg/dL)	0.75	±	0.2	−0.042		0.084	
eGFR (mL/min/1.73m^2^)	76	±	16	−0.111		0.165	*
⊿eGFR (mL/min/1.73m^2^/year)	−1.05	±	3.39	1		−0.187	*
Uric acid (mg/dL)	5.2	±	1.3	−0.125		0.033	
Systolic BP (mmHg)	128	±	12	−0.014		−0.051	
CV-Systolic BP (%)	8	±	22	−0.035		0.098	
Diastolic BP (mmHg)	72	±	1	0.003		0.112	
Urinary ACR (mg/g)	84	±	322	−0.208	**	0.067	
log ACR	1.30	±	0.6	−0.243	**	0.072	

*OHA* oral hypoglycemic agents, *CCB* calcium channel blockers, *RASi* renin-angiotensin system inhibitors, PG; plasma glucose, CV; coefficient of variation, *eGFR*; estimated glomerular filtration rate, ⊿eGFR; annual changes in eGFR, *BP* blood pressure, *ACR* albumin/creatinine ratio, *; *p <* 0.05,**; *p <* 0.01,***; *p <* 0.001

After the first visit in 2005 they were followed up in the subsequent at least 24 months through December 31, 2012 to assess kidney function with a median follow-up of 6.0 years (interquartile range; 4.1–6.5 years). Patients with hepatitis B surface antigen or antibodies against hepatitis C virus were excluded. Those who had aspartate aminotransferase and alanine aminotransferase of 100 U/L or greater, serum creatinine≧2.0 mg/dL were excluded as well. Information on smoking habits was collected through face-to-face interviews by TK. Smoking status was classified into one of three categories: current smokers, ex-smokers, and never smokers. Smokers in statistical analysis included current smokers (*n =* 52) and ex-smokers with the Brinkman index of 400 and higher (*n =* 5).

For each subject on each monthly visit, waist circumference, weight and BP were measured by registered nurses. BP using a sphygmomanometer after patients sat and rested for at least 5 min. Plasma glucose (PG), serum lipids and lipoproteins, creatinine, hepatic enzymes, uric acid and other blood tests were measured by standard methods using an autoanalyzer. HbA1C values were determined by high performance liquid chromatography and inter-assay CVs were between 2.0 and 3.0 %. LDL cholesterol was calculated using Friedewald’s formula in samples taken after an overnight fasting. Complete blood cell count was analyzed using an automated blood cell counter.

Urinary albumin was measured once during the first 3–4 months after enrollment in random urine samples using a turbidimetric immunoassay and expressed as albumin/creatinine ratio (ACR). Serum and urinary creatinine were measured enzymatically and estimated glomerular filtration rate (eGFR) was determined using the equation recommended by the Japanese Society for Nephrology [18].

Intrapersonal mean and coefficient of variation (CV) of HbA1c, fasting and post-breakfast plasma glucose (FPG and PPG, respectively) and serum triglycerides (FTG and PTG, respectively) taken during the first 12 months after enrollment were calculated in 168 patients with type 2 diabetes; 153 patients (91 %) had 12 measurements of HbA1c, systolic BP and 6 measurements of FPG, PPG, FTG and PTG, respectively. Linear regression was used to estimate changes in eGFR using a median of 52 creatinine measurements (interquartile range; 31-60) over 6.0 years of follow-up in each patient. Baseline means of serum creatinine and eGFR in Table 1 were means of 2–4 measurements during the first 3–4 months after enrollment.

Data were presented as mean ± SD unless otherwise stated. Differences between 2 groups were analyzed by t test and frequencies of conditions by Chi-square tests. Differences among 3 groups were analyzed using analysis of variance. Correlations of annual eGFR decline and CV-HbA1c were evaluated by Pearson correlation analysis. Stepwise multiple linear regression analyses were performed to further identify the most significant variables contributing to annual eGFR decline and CV-HbA1c. Potential confounders were forced into the model and standardized β coefficients were calculated. The explanatory power of the model was expressed as adjusted R^2^ values. A two-tailed *P* < 0.05 was considered statistically significant. All calculations were performed with SPSS system 15.0 (SPSS Inc., Chicago, IL).

## Results

Table 1 shows means of the intrapersonal mean values during the first 12 months after enrollment, except for age, duration of diabetes, serum creatinine, eGFR, ⊿eGFR and ACR. Means of age and duration of diabetes were those on enrollment of patients in the study. Baseline means of serum creatinine and eGFR in Table 1 were means of 2–4 measurements during the first 3–4 months after enrollment. ACR was measured once during the first 3–4 months after enrollment.

Patients had relatively good glycemic, lipid and BP control with a mean HbA1c of 7.0 %. CVs of HbA1c, FPG and PPG were 7.0 %, 14.1 % and 21.9 % respectively (Table 1). Baseline eGFR averaged 76 ± 16 ml/min/1.73m^2^ and eGFR change was linear and averaged -1.05 ± 3.39 ml/min/1.73m^2^ per year. Among 168 patients, 27 (16.0 %) had eGFR < 60 ml/min/1.73m^2^ and 53(31.5 %) had albuminuria (microalbuminuria 47, macroalbuminuria 6).

**Fig. 1:**
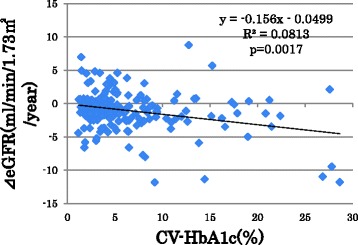
The scatter plot between annual changes in estimated glomerular filtration rate (⊿eGFR) and coefficient of variations (CV) of HbA1c

Changes in eGFR were inversely associated with CV-HbA1c (Fig. 1)、FTG、PTG、log ACR and smokers (Table 1). However, eGFR changes did not show significant associations with age, sex, duration of diabetes, baseline eGFR, treatment for diabetes, mean HbA1c and mean and CV of FPG, PPG and SBP.

Multiple linear regression analysis (Table 2) revealed that CV-HbA1c (standardized β、-0.257、*p =* 0.004) were associated with and log ACR (standardized β、-0.155、*p =* 0.085) and smoking (standardized β、-0.186、*p =* 0.062) tended to be associated with annual eGFR decline independently of age, sex, BMI, waist circumference, duration of and treatment for diabetes, means and CVs of FPG and PPG, mean HbA1c, baseline eGFR, FTG、PTG, and uses of anti-hypertensive and lipid-lowering medications.

**Table 2:** Multiple linear regression analysis for coefficient variation of HbA1c as a dependent variable

^Independent variables^	Standardized β	p values
sex	-.106	.267
age	-.101	.326
BMI	.061	.635
waist circumference	-.130	.316
duration of diabetes	-.047	.622
treatment for diabetes	-.121	.231
uses of anti-hypertensive medications	.002	.980
uses of lipid-lowering medications	.055	.537
smoking	-.186	.062
Fasting PG	.084	.488
Post-breakfast PG	.106	.376
HbA1c	.027	.812
CV-HbA1c	-.257	.004
Fasting TG	-.002	.989
Post-breakfast TG	-.072	.665
log ACR	-.155	.085
eGFR	-.074	.450

Abbreviations are the same as in Table

Patients were divided into 3 groups according to tertiles of CV-HbA1c (Table 3). As CV-HbA1c increased, the percentage of smokers, mean HbA1c, means and CVs of FPG and PPG, and TG increased whereas HDL cholesterol decreased. Diabetic patients in the highest as compared to the lowest and median tertiles of CV-HbA1c had faster annual decline in eGFR. Other parameters including baseline eGFR were not different among 3 groups.

**Table 3:** Anthropometric, clinical and biochemical features of patients with type 2 diabetes according to tertiles of CV-HbA1c

	CV-HbA1c tertiles									
	Low			Median			High			
	(1.17-3.64)			(3.64-6.50)			(6.50-28.65)			p values
Smokers (n, %)	10	,	18.2	22	,	39.3	25	,	44.6	0.008
Age (years)	63.6	±	9.6	62.3	±	9.4	61.2	±	11.3	0.453
BMI (kg/m^2^)	24.0	±	3.9	24.1	±	3.0	24.6	±	4.0	0.638
Waist circumference (cm)	87.2	±	9.4	86.6	±	8.7	87.0	±	11.5	0.959
Duration of diabetes (years)	9.2	±	7.7	10.6	±	6.7	9.8	±	7.5	0.622
Treatment of										
diabetes; diet/OHA/insulin (%)	43/50/7			32/45/23			20/ 57/ 23			0.026
hypertension; CCB/RASi/diuretics (%)	36/39/5			30/41/4			38/ 45/ 5			0.424
HbA1c (%)	6.6	±	0.6	7.1	±	0.7	7.4	±	1.0	<0.001
Fasting PG (mg/dL)	114	±	14	129	±	24	133	±	23	<0.001
Post-breakfast PG (mg/dL)	135	±	40	160	±	49	169	±	51	0.001
CV-HbA1c (%)	2.5	±	0.7	4.9	±	0.9	13.6	±	7.3	<0.001
CV-Fasting PG (%)	8.8	±	3.9	14.1	±	9.7	19.5	±	9.6	<0.001
CV-Post-breakfast PG (%)	18	±	10	23	±	11	25	±	11	0.007
Total cholesterol (mg/dL)	188	±	19	189	±	18	187	±	25	0.890
LDL cholesterol (mg/dL)	108	±	17	111	±	23	114	±	25	0.288
HDL cholesterol (mg/dL)	60	±	15	57	±	17	50	±	12	0.001
Fasting TG (mg/dL)	102	±	43	112	±	49	130	±	58	0.017
Post-breakfast TG (mg/dL)	131	±	59	144	±	65	137	±	65	0.047
Serum creatinine (mg/dL)	0.73	±	0.15	0.73	±	0.15	0.80	±	0.25	0.072
eGFR (mL/min/1.73m^2^)	74	±	12	76	±	15	77	±	21	0.641
⊿eGFR (mL/min/1.73m^2^/year)	−0.69	±	2.77	−0.63	±	2.29	−2.19	±	3.68	0.008
Uric acid (mg/dL)	5.3	±	1.5	5.0	±	1.4	5.2	±	1.1	0.495
Systolic BP (mmHg)	128	±	12	129	±	11	128	±	13	0.905
CV-Systolic BP (%)	8.2	±	2.3	7.6	±	2.1	8.3	±	2.2	0.254
Diastolic BP (mmHg)	72	±	6	72	±	7	72	±	7	0.787
Urinary ACR (mg/g)	21	±	24	69	±	152	162	±	532	0.066
log ACR	1.1	±	0.5	1.4	±	0.6	1.4	±	0.7	0.012

Mean ± SD or n, %. Abbreviations are the same as in Table 1

Association between HbA1c variability and renal function decline was significant in 53 patients with nephropathy (ACR≧30 mg/g) but did not reach statistical significance in 115 patients without　nephropathy (r = -0.400, *p =* 0.003 and r = -0.169, *p =* 0.07, respectively). Compared with patients with normoalbuminuria, annual eGFR declines were significantly faster in patients with microalbuminuria (ACR≧30 mg/g) after controlling for confounders described above (-2.0 ± 0.4 (SE) vs. -0.6 ± 0.3 ml/min/1.73m^2^ per year, *p =* 0.01).

## Discussion

Variations of HbA1c, a reflection of long-term glycemic fluctuation, were found to increase the risk of chronic kidney disease defines as estimated GFR (eGFR) <60 ml/min/1.73 m^2^ in some studies in patients with type 2 diabetes [14–16]. However, we are not aware of previous studies to determine whether HbA1c variability might directly associated with annual decline rate in eGFR in patients with type 2 as well as type 1 diabetes. The present study is the first to demonstrate a direct association between CV of HbA1c and annual eGFR decline in patients with diabetes independently of mean HbA1c and known predictors of GFR decline [19]. Further, association between HbA1c variability and renal function decline was stronger in patients with nephropathy (ACR≧30 mg/g) than in those with normoalbuminuria.

By comparison to short-term glucose variability, it has proven far less difficult to show an association between HbA1c variability and microvascular complication risk [20]. It has been shown that HbA1c variability predicted the development of chronic kidney disease in patients with type 2 diabetes [14, 16]. Further, Penno et al [15] have demonstrated that among 8260 patients with type 2 diabetes SD-HbA1c was associated with albuminuric chronic kidney disease independently of mean HbA1c and other known predictors of diabetic nephropathy, whereas mean HbA1c was not. These findings may be in line with our observation that albuminuria and CV-HbA1c were directly associated eGFR decline independently of mean HbA1c and other known predictors of GFR decline. However, among 4399 patients with type 2 diabetes in the intensive group of the ADVANCE trial [17], the association between SD of HbA1c and microvascular events did not reach statistical significance (*p =* 0.06 for trend) although there were significant linear associations of SD of HbA1c with combined macro—and microvascular events, major macrovascular events and all-cause mortality.

Although glycemic variability has been inconsistently associated with the risk of vascular complications in diabetes [21], several reasons may be involved in the association between visit-to-visit HbA1c variability and outcomes as suggested by Kilpatrick et al [20, 21]. They include ‘metabolic memory’ phenomenon [22]. They may be related to the fact that microvascular complication risk rises exponentially, rather than linearly, as HbA1c rises. They also may be related to the observation that acute improvement in HbA1c can lead to a short-term “early” worsening in retinopathy before subsequently resulting in a net long-term improvement. It is also possible that patient with HbA1c variability are those in whom the rest of their diabetes management is suboptimal.

Type 2 diabetic patients in the present study had annual eGFR decline which was even slower as compared with non-diabetic Japanese patients with early-stage chronic kidney disease (eGFR > 60 ml/min/1.73m^2^) [23] (-1.05 vs. -1.64 ml/min/1.73m^2^ per year). Further, annual eGFR decline of our patients was much slower than the rate found in a previous study of Japanese type 2 diabetic patients without clinical albuminuria (-2.94 ml/min/1.73m^2^ per year) [24] despite comparable baseline eGFR (76 and 75 ml/min/1.73m^2^). These findings may be due in part to the fact that our patients had better glycemic (mean HbA1c; 7.0 vs. 8.4 %) and BP (128/72 vs. 135/81 mmHg) control. Slower eGFR decline associated with better diabetic control in our patients may be related to failure to detect association between mean HbA1c and annual eGFR decline in the present study.

In the present study, patients with microalbuminuria had faster decline of eGFR than those with normoalbuminuria. This finding may be in line with previous studies that urinary albumin, even in the microalbuminuric range, is a predictor of renal function impairment in the general population [25], type 2 diabetic patients with preserved kidney function [26, 27] and in CKD patients (GFR < 50 ml/min) [28]. In the last-cited longitudinal observational study [28], Lorenzo et al. compared the rate of renal decline in diabetic and non-diabetic CKD patients with comparable levels of albuminuria. They found that urinary ACR was a robust predictor of poor outcome. In addition, the mean slope of renal decline was similar in diabetic and non-diabetic patients when controlling for albuminuria.

The strength of the current study is that we used a 1-year period when mean HbA1c and HbA1c variability were calculated from 12 measurements in 91 % participants. In addition, we measured serum creatinine and hence eGFR during follow-up period much more frequently than in previous studies [14–16]. This could contribute to the reliability of changes in kidney function. Such a testing frequency is routine in clinical settings in Japan. However, frequent measures of HbA1c may artificially inflate precision and decrease standard deviation, which may impact the results. Finally, BP control and variability and postprandial TG also have been taken into accounted. Major limitations are that study participants were small in number and from a single clinic in Japan. However, the characteristics of our study participants are similar to those reported in a previous large-scale study in Japan [29].

## Conclusions

The current study has shown direct association between HbA1c variability and kidney function decline in type 2 diabetic patients and demonstrated stronger association in patients with microalbuminuria than in patients with normoalbuminuria. These findings suggest that more attention should be paid by clinicians in diabetes control, avoiding excessive oscillations in blood glucose levels in type 2 diabetic patients in general and in those with microalbuminuria in particular. Further studies are needed to confirm the association in other ethnic groups with more patients.

## Abbreviations

ACR: Albumin /creatinine ratio
BP: Blood pressure
CV: Coefficient of variation
eGFR: Estimated glomerular filtration rate
FPG: Fasting plasma glucose
FTG: Fasting serum triglycerides
PPG: Post-breakfast plasma glucose
PTG: Post-breakfast serum triglycerides
